# Association of Free Sugar Intake Estimated Using a Newly-Developed Food Composition Database With Lifestyles and Parental Characteristics Among Japanese Children Aged 3–6 Years: DONGuRI Study

**DOI:** 10.2188/jea.JE20180036

**Published:** 2019-11-05

**Authors:** Aya Fujiwara, Kentaro Murakami, Keiko Asakura, Ken Uechi, Minami Sugimoto, Han-Chieh Wang, Shizuko Masayasu, Satoshi Sasaki

**Affiliations:** 1 Graduate School of Medicine, the University of Tokyo, Tokyo, Japan; 2 Interfaculty Initiative in Information Studies, the University of Tokyo, Tokyo, Japan; 3 School of Medicine, Toho University, Tokyo, Japan; 4 Faculty of Health Science, Toho University, Chiba, Japan; 5 Department of Social and Preventive Epidemiology, School of Public Health, the University of Tokyo, Tokyo, Japan; 6 Ikurien-naka, Ibaraki, Japan

**Keywords:** free sugar, food composition database, screen time, young children, Japan

## Abstract

**Background:**

The lack of comprehensive food composition databases for sugar contents in Japanese foods has led to the lack of nutritional epidemiologic studies on sugar intake in Japanese population. This cross-sectional study aimed to investigate the association of free sugar intake estimated using a newly developed food composition database with the characteristics and lifestyles of Japanese children aged 3–6 years.

**Methods:**

The food composition database contained information on sugars in 2,222 commonly consumed Japanese foods. Using this database, we estimated the sugar (total, added, and free sugars) intakes derived from a 3-day weighed dietary record of 166 boys and 166 girls aged 3–6 years living in 24 prefectures in Japan.

**Results:**

The mean free sugar intake was 26.8 g/d (standard deviation [SD], 12.3 g/d), while the mean value for energy intake was 7.8% (SD, 3.2%). The prevalence of excessive free sugar intake (≥10% of energy intake) was 21.7%. Among the characteristics and lifestyles examined, screen time was most strongly associated with the prevalence of excessive free sugar intake: multivariate adjusted odds ratios for screen time <0.5, ≥0.5 to <1, and ≥1 h/d were 1.0 (reference), 3.81 (95% confidence interval, 1.04–13.98), and 4.36 (95% confidence interval, 1.16–16.35), respectively. Additionally, younger age, shorter sleep, and mothers with office work and service and sales jobs (compared with those with professional and managerial jobs) were significantly associated with a higher prevalence of excessive free sugar intake.

**Conclusions:**

This study showed the sugar intake of Japanese children aged 3–6 years is positively associated with screen time.

## INTRODUCTION

Dietary habits during the early stages of life are associated with dietary intake^1^ and cardiovascular disease risk^2^ in the later stages of life. Therefore, development of healthy dietary habits in young children is essential for improving their health on a long-term basis. Studies in children suggested sugar intake is positively associated with dental caries,^3^ overweight,^4^ and cardiometabolic risk factors^5^ and contributes to excessive energy intake and inadequate micronutrient intake.^5^^–^^7^ Several countries have advocated the recommended^8^^–^^12^ and regulated^13^ intake of sugars that are not naturally occurring but are added during manufacture and cooking. The World Health Organization (WHO) provided recommendations on the intake of free sugar, including added sugar and sugars naturally occurring in fruit juices.^14^ Data related to the correlation of excessive sugar intake is needed to develop an effective intervention for the reduction of sugar intake.^15^ However, only a few nutritional epidemiologic studies on sugar intake have been performed in Japan.^16^^–^^20^ Thus, recommendations and regulations for sugar intake for children and adults were not advocated.^21^ as there was no available database for sugar contents of food items in Japan. In 2015, the sugar contents were reported for the first time in the Standard Table of Food Composition in Japan (STFCJ).^22^ Nevertheless, only 880 of the 2,222 food items in the STFCJ included sugar contents, without data available for free and added sugars.

Child-, parental-, and household-related factors, such as characteristics and lifestyles, were positively associates with excessive sugar intake (especially free or added sugar) among children in Western countries.^15^^,^^23^^–^^39^ While child-related factors (such as age) were positively associated with sugar intake,^15^^,^^23^^,^^26^^–^^31^^,^^33^^,^^35^^,^^38^^,^^39^ the associations between parental-related and household-related factors (such as socioeconomic status [SES]) remains inconsistent.^24^^–^^26^^,^^28^^–^^30^^,^^32^^,^^34^^,^^36^^,^^37^^,^^39^ More importantly, only a few studies have investigated the association between child-, parental-, and household-related factors and excessive sugar intake.^24^^,^^26^^,^^37^

Compared with Western children, Japanese children on average have a lower consumption of food groups contributing to free or added sugar intake^15^—sugars (as foods), confectionaries, dairy products, fruit juices, and sugar-sweetened beverages.^40^^–^^43^ Given these differences between Japanese and Western diets, the correlates of sugar intakes in Japanese children should be investigated.

This cross-sectional study investigated the association of free sugar intake estimated using a newly developed comprehensive sugar database with the characteristics and lifestyles of Japanese children aged 3–6 years.

## METHODS

### Study setting and participants

This cross-sectional analysis was based on the data derived from the Dietary Observation and Nutrient Intake for Good Hearth Research in Japanese young children (DONGuRI) study. We initially recruited 323 dietitians (referred to as research dietitians) working mainly in nursery schools in 24 prefectures in Japan. These research dietitians recruited children aged 1.5–6 years as study participants from nursery schools where the dietitians worked or related. Data collection was conducted between October and December 2015 by research dietitians in accordance with standard procedures.

**Figure 1.**
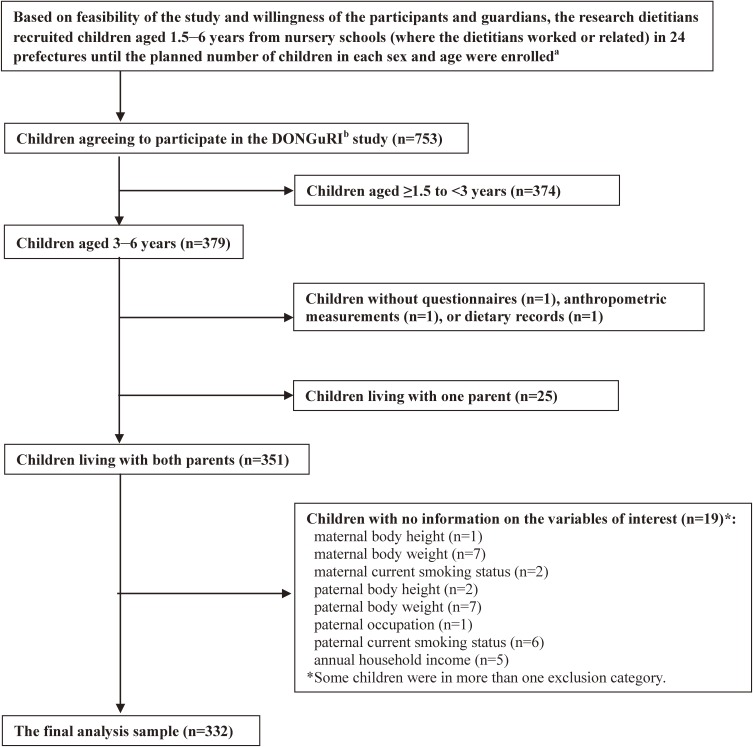
Eligibility for and participation in the present analysis (DONGuRI^b^ study). ^a^Two boys and two girls aged 3, 4, 5, and 6 year as well as eight boys and eight girls aged ≥1.5 to <3 years in each prefecture. ^b^DONGuRI: Dietary Observation and Nutrient intake for Good hearth Research In Japanese young children.

In each prefecture, two boys and two girls aged 3, 4, 5, and 6 years (16 children in total) were recruited, as well as eight boys and eight girls aged ≥1.5 to <3 years (the target number of study participants was 768 in total). Excluded from the study were participants under diet therapy as ordered by a doctor or dietitian at the time of the study, having particular dietary habits (such as vegetarian), planning to move until March 2016, and having guardians who are dietitians or medical doctors. Recruitment was conducted based on feasibility of the study and willingness of the participants and guardians until the planned number of children in each sex and age (as mentioned above) were enrolled. This study was thus conducted based on a voluntary basis, including 753 children from 315 nursery schools (Figure 1). Dietary intake in children aged 3–6 years was assessed using a 3-d dietary record (DR), that in children aged <3 years was assessed using a 1-d DR; thus, the present analysis included only children aged 3–6 years (*n* = 379). After excluding children without questionnaire-based data, anthropometric measurements, or DR data, we restricted our analysis to 351 children living with both parents to investigate the effects of both maternal and paternal characteristics on sugar intake. We then excluded 19 children with no variables of interest. The final analysis sample comprised 332 children.

This study was conducted according to the principles of the revised Declaration of Helsinki, and all procedures involving human subjects were approved by the Ethics Committee of the University of Tokyo, Faculty of Medicine (approval number: 10885; approval date: 27 July 2015). The research dietitians explained the aims and procedure of the study to all guardians of children. A written informed consent was obtained from all children and their guardians.

### Dietary assessment

The research dietitians and the guardians were asked to record children’s dietary intake using non-consecutive, 3-d DR, which consisted of 2 weekdays (with a lunch at the nursery schools) and 1 weekend day (without a lunch at the nursery schools). Length of days to complete 3-d DR was ranged from 3 to 39 days (mean: 9.2 days) because DRs of 23 children (6.9%) were not non-consecutive. The research dietitians and the guardians were given recording sheets for the DR (as well as questionnaires designed for present study) and asked to conduct DR after the guardians answered the questionnaires. The research dietitians were asked to weigh and record all foods and drinks that the children consumed on survey days at the nursery schools as well as the amount of leftovers. They were encouraged to provide as much information as possible, including the names of dishes, foods, and ingredients, and whether the foods were prepared at nursery schools or were ready made. When the dietitians encountered difficulties in weighing (≤20% of all the cases), the staff in the nursery schools were then asked to take the weight instead.

The guardians were asked to weigh and record all foods and drinks the children consumed as well as the amount of leftovers outside the nursery schools on survey days. The guardians were provided a digital kitchen scale (KD-812WH; Tanita, Tokyo, Japan), a measuring spoon, a measuring cup, and a handbook for the DR. The research dietitian orally explained the recording procedure to the guardians. When the guardians encountered some difficulties in weighing (eg, eating out), they were asked to record the estimated amount of foods and drinks consumed in addition to the restaurant’s name. On the following school day of each recording day, recording sheets as well as packages of processed foods were directly handed to the dietitians and were checked by them. If the dietitians found missing or unclear information, they collected more information from the guardians.

The research dietitians then assigned food codes for all food items in both recording sheets according to STFCJ.^22^^,^^44^ Moreover, the dietitians estimated the amount (g) of foods and drinks consumed by the children based on the data provided. Subsequently, the recorded food items and weight were reconfirmed by two dietitians at the central office. Intakes of energy and selected nutrients were calculated according to STFCJ.^22^^,^^44^

### Development of food composition database

**Figure 2.**
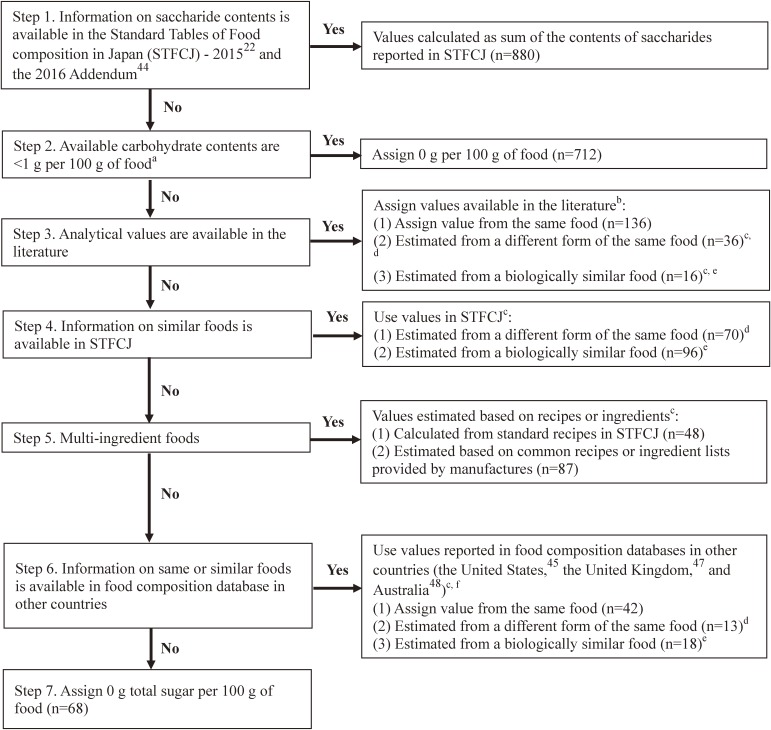
Flow chart for the development of a total sugar database. ^a^Available carbohydrate contents were calculated as subtracting dietary fiber content (g/100 g of food) from carbohydrate. ^b^A search strategy for the literature is described in online supplement. ^c^Values were adjusted using the ratio of the dry weight between interested and referred food items. ^d^Total sugar content of the cooked or dried food was calculated from the raw form. ^e^Values from a similar food item (same family or order) were assigned after comparing total energy and other nutrient contents. ^f^When the value of a specific food item was available in several countries, the source of imports was considered primary and a similarity for total energy and other nutrient contents was a second consideration. When assigning data from the United Kingdom, disaccharide values were multiplied by 0.95 since values were shown as monosaccharide equivalents.^47^

A comprehensive food composition database for sugars in 2,222 Japanese food items was recently developed to calculate sugar intakes (the database is available upon request). Details on how the database was established are available in the online supplement. Briefly, the United States Department of Agriculture (USDA) defined total sugar as the sum of all mono- and disaccharides, including glucose, fructose, galactose, sucrose, lactose, and maltose.^45^ Total sugar content in each food was determined using a seven-step method. This method includes a stepwise strategy in data gathering, as proposed by Rand et al,^46^ based on the saccharide contents in STFCJ^22^^,^^44^ (Steps 1, 2, 4, and 5); literature (search strategy is available in the online supplement) measuring the sugar content in foods (Step 3), and food composition tables in foreign countries^45^^,^^47^^,^^48^ (Step 6), as well as assigning 0 g total sugar to the remaining foods (Step 7) (Figure 2 and Table 1). Added sugar was defined as sugars and syrups added to food during processing or preparation, excluding naturally occurring sugars in foods.^49^ Free sugar was defined as all mono- and disaccharides added to foods and beverages by the manufacturer, cook or consumer, and sugars naturally presenting in honey, syrups, fruit juices, and fruit juice concentrate.^14^ Contents of added and free sugars in all food items were determined using a published stepwise method^50^ based on the contents of total sugar and saccharides (Table 2).

**Table 1.** Number of food items in each step in the development of a total sugar database

Food groups^a^	Steps^b^							Total

	1	2	3	4	5	6	7	
Cereals and potatoes	190	4	15	11	1	0	5	226
Sugars and jams	30	0	0	0	6	0	0	36
Pulses and nuts	98	1	6	14	3	1	6	129
Fruits and vegetables^c^	288	74	74	115	7	12	34	604
Protein rich animal foods^d^	69	573	15	3	68	42	17	787
Confectionaries	132	0	20	5	6	0	0	163
Fruit and vegetable juices	14	0	2	2	1	0	0	19
Sugar-sweetened beverages^e^	7	0	23	0	2	0	0	32
Seasonings	34	14	23	7	35	15	4	132
Other foods^f^	18	46	10	9	6	3	2	94
Total	880	712	188	166	135	73	68	2,222

^a^Food groups were defined based on the culinary usage and the similarity of nutrient profiles of the foods, mainly according to the Standard Tables of Food composition in Japan (STFCJ)- 2015^22^ and the 2016 Addendum.^44^

^b^Step 1: assign values available in STFCJ^22^^,^^43^; step 2: assign 0 g per 100 g of food to foods with <1 g available carbohydrates (calculated as subtracting dietary fiber content (g per 100 g of food) from carbohydrate) per 100 g; step 3: assign analytical values reported in the literature; step 4: use values of similar foods available in STFCJ^22^; step 5: estimated based on recipes or ingredients; step 6: use values from food composition databases in other countries (the United States,^45^ the United Kingdom,^47^ and Australia^48^); step 7: assign 0 g per 100 g of food.

^c^Including mushrooms and seaweeds.

^d^Consisting of fish, meats, eggs, milk (except for milk beverages), and dairy products.

^e^Consisting of soda, sports drinks, fruit drinks, milk beverages, and pre-sweetened tea and coffee.

^f^Consisting of fat and oil, alcoholic beverages (added during cooking or processing), unsweetened tea and coffee, and ready-made curry and stew.

**Table 2.** Number of food items in each step in the development of added or free sugar database

Food groups^a^	Steps^b,c^											Total

	1	2	3	4	5	6	7	8	9	10	11	
Cereals and potatoes	37	159	2	3	0	1	0	18	4	2	0	226
Sugars and jams	0	0	27	5	0	0	1	2	1	0	0	36
Pulses and nuts	6	108	3	2	3	0	0	4	0	2	1	129
Fruits and vegetables^d^	119	408	2	22	9	0	0	31	1	7	5	604
Protein rich animal foods^e^	607	67	101	2	0	3	0	0	3	0	4	793
Confectionaries	4	6	103	22	0	13	0	3	11	1	0	163
Fruit and vegetable juices	0	19	0	0	0	0	0	0	0	0	0	19
Sugar-sweetened beverages^f^	0	0	5	0	0	6	0	21	0	0	0	26
Seasonings	20	28	6	3	3	1	0	32	29	10	0	132
Other foods^g^	50	23	0	3	0	0	2	8	4	4	0	94
Total	843	818	249	62	15	24	3	119	53	26	10	2,222

^a^Food groups were defined based on the culinary usage and the similarity of nutrient profiles of the foods, mainly according to the Standard Tables of Food composition in Japan (STFCJ) - 2015^22^ and the 2016 Addendum.^44^

^b^Added sugar contents were defined as follows: step 1: assign 0 g per 100 g of food to foods with 0 g total sugar per100 g; step 2: assign 0 g per 100 g of food to no added sugar food groups (eg, plain cereals [such as grains, breads, pastas, rice, and flours], plain nuts and pulses, fresh fruits and vegetables, fresh meat and seafood, egg, non-sweetened dairy products, fats and oils, 100% fruit and vegetable juices, non-sweetened coffee and tea, and non-sweetened alcoholic beverages); step 3: assign values of total sugar to 100% added sugar food groups (eg, sugar and syrups; processed meats; confectioneries not containing fruits, dairy products, and chocolates; soft drinks except for fruit drinks, and bouillon cubes); step 4: calculated based on standard recipes available in STFCJ^22^ to foods whose ingredients were all assigned in steps 1–3; step 5: calculated based on unsweetened variety; step 6: Estimated from content of each saccharide (ie, calculated as subtracting lactose content from total sugar content for sweetened dairy products and confectionaries containing daily products); step 7: use values from food composition databases in other countries (Australia^48^ [*n* = 1] and Denmark^66^ [*n* = 2]); step 8: calculated based on common recipes or ingredients; step 9: calculated based on standard recipes available in STFCJ^21^ for foods with ingredient values that were assigned in from steps 5–8; step 10: assign a half of total sugar content; step 11: assign 0 g per 100 g of food.

^c^Free sugar contents were estimated as sum of added sugar contents and total sugar contents from fruit juices.

^d^Including mushrooms and seaweeds.

^e^Consisting of fish, meats, eggs, milk (except for milk beverages), and dairy products.

^f^Consisting of soda, sports drinks, fruit drinks, milk beverages, and pre-sweetened tea and coffee.

^g^Consisting of fat and oil, alcoholic beverages (added during cooking or processing), unsweetened tea and coffee, and ready-made curry and stew.

### Assessment of characteristics and lifestyles

All the information was obtained using questionnaires designed for this study, unless otherwise indicated. The questionnaires were collected during the study period by the research dietitians and were sent to the central office. Body height (to 0.1 cm) and weight (to 0.1 kg) of the children were measured while the children were wearing light clothing with no shoes, as part of this study or a routine health check-up (within 1 month before the study period). Weight status was defined according to the International Obesity Task Force age- and sex-specific body mass index (BMI) (kg/m^2^) cut-offs.^51^ Children with BMI values that corresponded to an adult BMI of <18.5 kg/m^2^ were classified as underweight, ≥18.5 to <25 kg/m^2^ as normal, and ≥25 kg/m^2^ as overweight and obese. Sleeping duration was defined as the sum of daytime naps at nursery schools (reported by research dietitians or staff of nursery schools) and night-time sleep (reported by guardians) and categorized as <10 or ≥10 h/d according to the recommendations of American Academy of Sleep Medicine.^52^ Outdoor playtime was defined as the duration of outdoor playtime at nursery schools on weekdays (reported by research dietitians or staff of nursery schools) and weekend days (reported by guardians). The number of weekdays (5/7) and weekend days (2/7) per week were calculated, and the results were classified into <1, ≥1 to <2, or ≥2 h/d. Children’s screen time, defined as the amount of time watching TV and playing video games, for weekdays and weekend days was reported separately by their guardians; after calculating the number of weekdays and weekend days, the results were categorized as <0.5, ≥0.5 to <1, or ≥1 h/d.

The guardians were asked to report the characteristics of parents (age [20 to 29, 30 to 39, or ≥40 years], body height and weight, educational level [≤12, 13 to 14, or ≥15 years], occupation, and current smoking status [yes or no]) and household (living with grandparent [s] [yes or no], number of siblings [0, 1, or ≥2], and annual income). Parents’ body weight was defined based on the BMI recommended by the WHO: underweight (<18.5 kg/m^2^), normal (≥18.5 to <25 kg/m^2^), and overweight and obese (≥25 kg/m^2^).^53^ Parental occupation was categorized as professional and managerial job, office work and service/sales, manual (including farming/forestry/fishery, transportation, labour service, and others), or unemployed.^54^ Annual household income was adjusted by household size and composition^55^ and classified into approximate tertiles: low (<2,380,000 yen/year), middle (≥2,380,000 to <3,340,000 yen/year), or high (≥3,340,000 yen/year).

### Statistical analysis

Excessive free sugar intake was defined as ≥10% of the total energy intake recommended by the WHO.^14^ Intakes of energy and energy-providing nutrients are presented as mean and standard deviation (SD). In addition to total intake, the intake of free sugar from each food group was examined. Food groups were defined based on the culinary usage and similarity of nutrient profiles, mainly according to STFCJ.^22^^,^^44^ Differences in dietary intake between participants consuming free sugar excessively and those consuming adequate amounts of free sugar were analyzed using an independent *t*-test. The prevalence of excessive free sugar intake was estimated using logistic regression. First, the crude odds ratios (ORs) and 95% confidence intervals (CIs) for the prevalence of excessive free sugar intake were calculated based on the lifestyle patterns of children and the following characteristics, which could be associated with excessive free sugar intake: sex (reference: boys), age (reference: 3 years), weight status (reference: normal), sleeping duration (reference: <10 h/d), outdoor playtime (reference: <1 h/d), and screen time (reference: <0.5 h/d) of children; parental age (reference: 20 to 29 years), weight status (reference: normal), educational level (reference: ≤12 years), occupation (reference: professional and manager), and current smoking status (reference: no); living status of grandparent(s) (reference: no); number of siblings (reference: 0); and household income (reference: low). The multivariate-adjusted ORs and 95% CIs were calculated by entering all the variables simultaneously into the regression model. All analyses were performed using SAS version 9.4 (SAS Institute, Cary, NC, USA). All *P* values reported are two sided, and *P* values of <0.05 were considered statistically significant.

## RESULTS

This analysis included 332 children (166 for both sexes) with a mean age of 4.4 (SD, 1.1) years. The average free sugar intake was 26.8 (SD, 12.3) g/d (Table 3). The food groups that contributed to free sugar intake included confectionaries (mean: 34.5%), sugar-sweetened beverages (18.4%), sugars and jams (18.2%), seasonings (11.3%), and fruit and vegetable juices (6.2%). The percentage of energy from free sugar was 7.8% (SD, 3.2%), and the prevalence of excessive free sugar intake (≥10% of energy intake) was 21.7%. Excessive free sugar intake was mainly derived from confectionaries, sugar-sweetened beverages, and fruit and vegetable juices. While there was no significant difference in energy intake between individuals with and without excessive free sugar intake, those with excessive free sugar intake had a lower mean energy-adjusted intake of protein and higher mean energy-adjusted intakes of carbohydrates and total, added, and free sugars, with no difference in total and saturated fat intakes.

**Table 3.** Dietary intake in Japanese children aged 3–6 years (*n* = 332)

	Total (*n* = 332)		Participants with excessive free sugar intake^a^ (*n* = 72)		Participants without excessive free sugar intake^b^ (*n* = 260)		*P*^c^
		
	Mean	SD	Mean	SD	Mean	SD	
Total free sugar, g/d	26.8	12.3	43.1	10.1	22.3	8.5	<0.0001
Free sugar from each food group, g/d							
Cereals and potatoes	0.5	1.0	0.6	1.2	0.4	1.0	0.30
Sugars and jams	3.9	3.2	4.3	3.8	3.8	3.1	0.25
Pulses and nuts	0.1	0.5	0.1	0.5	0.1	0.5	0.88
Fruits and vegetables^d^	0.3	0.7	0.4	1.1	0.3	0.6	0.21
Protein rich animal foods^e^	1.3	1.7	2.0	2.8	1.1	1.2	0.009
Confectionaries	10.3	7.4	16.9	8.9	8.5	5.8	<0.0001
Fruit and vegetable juices	2.2	4.1	4.3	5.8	1.6	3.2	0.0002
Sugar-sweetened beverages^f^	6.0	6.6	12.0	8.8	4.3	4.6	<0.0001
Seasonings	2.0	1.1	1.9	0.9	2.0	1.1	0.48
Other foods^g^	0.3	1.0	0.4	1.5	0.2	0.8	0.35
Energy, kcal/d	1371	231	1397	222	1364	233	0.29
Energy-providing nutrient, % of energy							
Protein	14.2	1.5	13.6	1.4	14.3	1.5	<0.0001
Total fat	29.2	4.0	28.4	3.5	29.4	4.1	0.052
Saturated fatty acid	9.6	1.9	9.6	1.9	9.5	2.0	0.69
Carbohydrate	55.7	4.5	57.3	4.1	55.2	4.5	0.0004
Total sugar	16.3	3.9	20.8	3.3	15.1	3.0	<0.0001
Added sugar	6.9	2.9	10.7	2.6	5.9	2.0	<0.0001
Free sugar	7.8	3.2	12.4	2.3	6.5	2.1	<0.0001

SD, standard deviation.

^a^Defined as participants consuming ≥10% of energy from free sugar according to World Health Organization recommendations.^14^

^b^Defined as participants consuming <10% of energy from free sugar according to World Health Organization recommendations.^14^

^c^Difference between participants with excessive and adequate free sugar intake was tested using independent *t*-test.

^d^Including mushrooms and seaweeds.

^e^Consisting of fish, meats, eggs, milk (except for milk beverages), and dairy products.

^f^Consisting of soda, sports drinks, fruit drinks, milk beverages, and pre-sweetened tea and coffee.

^g^Consisting of fat and oil, alcoholic beverages (added during cooking or processing), unsweetened tea and coffee, and ready-made curry and stew.

In univariate analyses, only screen time among the selected characteristics and lifestyles was positively associated with excessive free sugar intake (Table 4). When all the variables were entered simultaneously, screen time remained positively associated with excessive free sugar intake: the prevalence in children watching TV and playing video game ≥0.5 to <1 h/d and ≥1 h/d were almost four times as high as the prevalence in children with >0.5 h/d screen time. Meanwhile, the prevalence of excessive free sugar intake was negatively associated with age of children: the prevalence of excessive free sugar intake in 6-year-olds was one-third of that in 3-year-olds. In addition, the prevalence of excessive free sugar intake in children sleeping more than 10 h/d was 40% compared with that of their counterparts. The prevalence of excessive free sugar intake in children with mothers engaging in office jobs and service/sales was almost twice as high as the prevalence in children with mothers engaging in professional and managerial jobs. In contrast, excessive free sugar intake had no significant association with other characteristics and lifestyles of children, parents, and households.

**Table 4.** Associations between selected characteristics and lifestyles and excessive free sugar intake in Japanese children aged 3–6 years (*n* = 332)

	*n* of total participants	*n* with excessive intake^a^	Crude model	Multivariate model^b^
	
			OR (95% CI)	OR (95% CI)
Children’s characteristics and lifestyles				
Sex				
Boys	166	37	1.0 (ref)	1.0 (ref)
Girls	166	35	0.93 (0.55, 1.57)	0.91 (0.49, 1.67)
Age, years				
3	87	23	1.0 (ref)	1.0 (ref)
4	86	17	0.69 (0.34, 1.40)	0.61 (0.27, 1.38)
5	85	18	0.75 (0.37, 1.51)	0.43 (0.18, 1.04)
6	74	14	0.65 (0.31, 1.38)	0.36 (0.13, 0.94)
Weight status^c^				
Underweight	35	8	1.06 (0.46, 2.44)	0.83 (0.31, 2.19)
Normal	269	59	1.0 (ref)	1.0 (ref)
Overweight and obese	28	5	0.78 (0.28, 2.12)	0.86 (0.27, 2.72)
Sleeping duration, hours/d				
<10	52	15	1.0 (ref)	1.0 (ref)
≥10	280	57	0.63 (0.32, 1.23)	0.41 (0.18, 0.94)
Outdoor playtime, hours/d				
<1	85	21	1.0 (ref)	1.0 (ref)
≥1 to <2	158	36	0.90 (0.49, 1.67)	0.79 (0.39, 1.61)
≥2	89	15	0.62 (0.29, 1.30)	0.67 (0.27, 1.62)
Screen time, hours/d				
<0.5	44	3	1.0 (ref)	1.0 (ref)
≥0.5 to <1	173	39	3.98 (1.17, 13.54)	3.81 (1.04, 13.98)
≥1	115	30	4.82 (1.39, 16.73)	4.36 (1.16, 16.35)
Maternal characteristics				
Age, years				
20 to 29	20	4	1.0 (ref)	1.0 (ref)
30 to 39	224	42	0.92 (0.29, 2.90)	2.08 (0.24, 18.31)
≥40	88	26	1.68 (0.51, 5.50)	4.60 (0.45, 46.68)
Weight status^d^				
Underweight	47	9	0.82 (0.37, 1.79)	0.77 (0.30, 1.95)
Normal	258	58	1.0 (ref)	1.0 (ref)
Overweight and obese	27	5	0.78 (0.28, 2.16)	0.65 (0.21, 1.99)
Educational level, years				
≤12	63	15	1.0 (ref)	1.0 (ref)
13 to 14	153	37	1.02 (0.51, 2.03)	1.37 (0.56, 3.37)
≥15	116	20	0.67 (0.31, 1.42)	0.85 (0.32, 2.32)
Occupation				
Professional and manager	162	28	1.0 (ref)	1.0 (ref)
Office work, service, and sales	129	34	1.71 (0.97, 3.01)	2.14 (1.08, 4.25)
Manual^e^	28	6	1.31 (0.49, 3.51)	1.53 (0.47, 4.97)
Unemployed	13	4	2.13 (0.61, 7.40)	2.50 (0.60, 10.37)
Current smoking status				
No	308	65	1.0 (ref)	1.0 (ref)
Yes	24	7	1.54 (0.61, 3.87)	1.78 (0.58, 5.44)
Paternal characteristics				
Age, years				
20 to 29	12	3	1.0 (ref)	1.0 (ref)
30 to 39	198	38	0.71 (0.18, 2.76)	0.42 (0.03, 5.39)
≥40	122	31	1.02 (0.26, 4.02)	0.52 (0.04, 7.70)
Weight status^d^				
Underweight	12	3	1.23 (0.32, 4.73)	0.81 (0.18, 3.70)
Normal	235	50	1.0 (ref)	1.0 (ref)
Overweight and obese	85	19	1.07 (0.59, 1.94)	0.74 (0.37, 1.49)
Educational level, years				
≤12	113	23	1.0 (ref)	1.0 (ref)
13 to 14	76	20	1.40 (0.70, 2.78)	1.30 (0.57, 2.95)
≥15	143	29	1.00 (0.54, 1.84)	1.36 (0.57, 3.23)
Occupation				
Professional and manager	148	34	1.0 (ref)	1.0 (ref)
Office work and service/sales	90	17	0.78 (0.41, 1.50)	0.58 (0.26, 1.25)
Manual^e^	92	21	0.99 (0.53, 1.84)	1.01 (0.48, 2.15)
Unemployed	2	0	—	—
Current smoking status				
No	186	43	1.0 (ref)	1.0 (ref)
Yes	146	29	0.82 (0.49, 1.40)	0.74 (0.39, 1.42)
Household’s’ characteristics				
Living with grandparent(s)				
No	272	56	1.0 (ref)	1.0 (ref)
Yes	60	16	1.40 (0.74, 2.67)	1.95 (0.90, 4.19)
Number of siblings				
0	54	14	1.0 (ref)	1.0 (ref)
1	182	39	0.78 (0.39, 1.58)	1.42 (0.61, 3.28)
≥2	96	19	0.71 (0.32, 1.55)	0.78 (0.29, 2.06)
Income				
Low (<2,380,000 yen/year)	91	25	1.0 (ref)	1.0 (ref)
Middle (≥2,380,000 to <3,340,000 yen/year)	144	28	0.64 (0.34, 1.18)	0.48 (0.22, 1.01)
High (≥3,340,000 yen/year)	97	19	0.64 (0.33, 1.27)	0.45 (0.19, 1.07)

CI, confidence interval; OR, odds ratio; Ref., reference standard.

^a^Defined based on percentage of energy intake from free sugar according to World Health Organization recommendations^14^: ≥10% for excessive intake.

^b^All the variables listed were entered into the model simultaneously.

^c^Defined according to the International Obesity Task Force age- and sex-specific body mass index (calculated as kg/m^2^) cutoffs, which correspond to an adult body mass index of <18.5 for underweight, ≥18.5 to <25 for normal, and ≥25 for overweight and obese subjects.^51^

^d^Defined based on body mass index (calculated as kg/m^2^) according to World Health Organization recommendations^53^: <18.5 for underweight, ≥18.5 to <25 for normal, and ≥25 for overweight and obese subjects.

^e^Including farming/forestry/fishery, transportation, labour services, and other.

## DISCUSSION

To our knowledge, the present study is the first to estimate free sugar intake and identify its association with selected characteristics and lifestyles in a non-Western country. Several Japanese studies estimated the sugar intake in children^16^^–^^18^ and adults.^19^^,^^20^ However, these sugar intakes were estimated based on the restricted forms of sugar (glucose, fructose, and sucrose)^16^ or the restricted food sources (mainly sugars, confectionaries, and beverages).^17^^–^^20^ It was not indicated in the estimation if the amount consumed was an added sugar or a naturally occurring sugar.^16^^–^^20^ Here, we estimated the intake of free, added, and total sugar by developing a comprehensive database that contains data on the sugar contents of various food items, and the results will help estimate the prevalence of excessive intake as well as the main dietary sources of these sugars in different Japanese populations.

As expected, the mean value of free sugar intake and the prevalence of excessive free sugar intake among Japanese children were lower than those in Western countries. The four national representative surveys from Western countries reported that energy-adjusted free sugar intake in children ranged from 12% to 21%^29^^,^^31^^,^^34^^,^^38^ and at least 70% of children had excessive free sugar intake (≥10% of energy intake).^29^^,^^34^^,^^38^ Regarding added sugar (the amount is estimated by subtracting sugar present in fruit juices from free sugar), the eight national representative surveys from Western countries showed that the energy-adjusted intake of children was more than 9.0% of the total energy intake.^5^^,^^23^^,^^27^^,^^34^^,^^38^ These differences in free sugar intake between Western and Japanese children were due to differences in food consumption patterns. The consumption of confectionaries, sugar-sweetened beverages, and fruit juices (mean: 64, 68, and 27 g/d, respectively) in Japanese children was on average lower than those of Western children (mean: 85, 313, and 93 g/d in the United Kingdom^41^; 53, 197, and 73 g/d in Ireland^42^; and 118, 140, and 120 g/d in Australia,^43^ respectively), although these food groups contributed to the higher free sugar intake reported in this study as well as those in Western countries.^29^^,^^31^^,^^38^

Among the child-related factors examined, longer screen time and short sleep were associated with excessive free sugar intake among Japanese children. This observation is in agreement with the results of the previous Western studies conducted among schoolchildren, which indicated associations between lifestyles and added sugar intake.^39^^,^^56^ The underlying mechanisms for these associations are unclear. One possible reason is the influence of food advertising on TV, which promotes the consumption of sugar-rich foods, such as sugar-sweetened beverages.^57^ With regard to sleep duration, Kjeldsen et al^56^ suggested that changes in reward-related brain functions due to a shorter sleep duration increases the individual’s drive to consume sugar-containing foods. Moreover, since both longer screen time and short sleep can be considered unhealthy lifestyles, the excessive free sugar intake in children with these lifestyles may show that parents less likely encourage their children to practice a healthy lifestyle, including following the recommended dietary intake. Contrary to the results of the previous Western studies,^15^^,^^23^^,^^26^^,^^28^^–^^31^^,^^33^^,^^35^^,^^38^^,^^39^ a negative association between age and free sugar intake was observed in the present study. Nevertheless, it should be noted that most Western studies investigated children and adolescents with a wide age range (eg, 4–18 years), while the participants were 3–6 years old in this study. Compared with Western countries, however, younger children in Japan should avoid the excessive consumption of free sugar.

Among the SES variables investigated, the only factor associated with lower free sugar intake was maternal engagement with highly skilled occupation in the present study. The associations between SES variables and children’s sugar intake reported in Western studies are far from consistent, with positive,^26^^,^^28^ inverse,^30^^,^^32^^,^^34^^,^^36^^,^^37^^,^^39^ null,^24^^,^^25^^,^^29^^,^^36^^,^^37^ and even inverted U-shaped^26^ associations. Although inconsistency may be due to differences in the choice and definition of SES variables (eg, employment,^26^ social class,^29^^,^^32^ education,^24^^,^^26^^,^^30^^,^^36^^,^^37^^,^^39^ income,^25^^,^^26^^,^^34^^,^^36^ and overall SES index^28^), population characteristics, dietary assessment method, and sugar intake level and distribution as well as sample size, these results may reflect the true association in each population, suggesting the need for country-specific research.

This study had several limitations. First, while only about 50% of the children aged 3–6 years attend nursery school in Japan,^58^ the study participants were recruited from nursery school. Therefore, the study participants were not a representative sample of Japanese children. In addition, the study participants had highly educated and skilled parents and lived with more siblings than general Japanese children.^59^^,^^60^ Nevertheless, other parental and household characteristics (mean height and weight, living status of grandparent(s), and household income),^40^^,^^60^ as well as the mean values of children’s height and weight by sex and age,^61^ were comparable to that in general Japanese children. Second, we restricted our analysis to children living with both parents because we are interested in the effect of both maternal and paternal variables. Thus, the children analyzed in the present study were from two-parent households and the present results may not be applicable to single-parent households. However, the association between household structure and dietary intake is an important issue for children. In previous Western studies, children with a single parent had unhealthy dietary habits (more sugar-sweetened beverages^57^ and fewer fruits and vegetables^62^) compared to children with two parents. Further research is needed for dietary intake of these vulnerable populations in Japan. Third, the accuracy of data on the characteristics and lifestyles of the study participants obtained from questionnaires remain unknown. However, it cannot be assumed that the accuracy of the response to questionnaires depends on free sugar intake of the participants. Fourth, while DRs contain detailed information on individual diet, this method is self-reported and measurement errors may occur. However, it is generally acknowledged that data on the dietary intake of young children reported by their parents is more accurate than those of other ages, since the dietary intake of young children is highly supervised by their parents.^63^ Moreover, errors when measuring the energy-adjusted intakes should be minimised in this study.^64^ Finally, several unavoidable limitations occurred when the sugar dataset was developed. Although, a total of 1,592 food items were determined using saccharide contents (*n* = 880) or available carbohydrate contents (*n* = 712) in STFCJ,^22^^,^^44^ the sugar contents of other items were determined using the values of similar food items (*n* = 239) or recipes (*n* = 135). This procedure may lead to the under- or overestimation of sugar intake because we could not consider the difference in food items among biologically similar foods and the changes occur during cooking and processing.^50^ In addition, the variation of sugar contents in commercial foods^65^ was a possible cause of under- or overestimation of sugar intake although the variation of nutrient composition (for not only sugar but also other nutrients) is a general limitation in dietary surveys. This is because the food composition table may not necessarily reflect the true nutrient composition of the foods. Nevertheless, we did not examine the reliability of the database by comparing estimated total sugar intake with the values measured using analytical methods (eg, duplicated method) or biomarkers (eg, urinary sugar excretion) in the present study. Thus, the accuracy of the estimated total sugar intake remained unknown. For free and added sugar, comparison of the estimated intake with the values measured using analytical methods or biomarkers is impossible because free sugar (as well as added sugar) and naturally occurring sugar do not differ chemically. In any case, further research is needed on sugar contents for food items without values in the STFCJ as well as research using duplicated methods or biomarkers to accurately estimate sugar intake in Japanese population.

In conclusion, this cross-sectional study conducted in Japanese children aged 3–6 years reported the sugar intakes and the prevalence of excessive free sugar intake (≥10% of energy) using a newly developed database and identified several lifestyles and characteristics associated with excessive sugar intake, including longer screen time, younger age, shorter sleep, and maternal engagement with office work, service, and sales. Thus, children with these lifestyles and characteristics should be potential targets of free sugar reduction when such a reduction campaign will be planned and conducted in Japan in the future. As the prevalence of children with excessive free sugar intake was relatively high (21.7%) in this Japanese population, the next step is to clarify the association of free sugar intake with nutrient intake and health status in Japanese children.

## APPENDIX A. SUPPLEMENTARY DATA

eAppendix 1. Development of sugar content database
