# Supplementary material for: Association of Free Sugar Intake Estimated Using a Newly-Developed Food Composition Database With Lifestyles and Parental Characteristics Among Japanese Children Aged 3–6 Years: DONGuRI Study
Source: J Epidemiol. 2019 Nov 5;29(11):414–23. doi: 10.2188/jea.JE20180036 (PMC6776475; doi:10.2188/jea.JE20180036)
Supplement: Supplementary file 1 [file je-29-414-s001.pdf]

eAppendix 1. Development of sugar content database

A sugar content database was developed based on Standard Tables of Food Composition in Japan (STFCJ) - 2015[1] and the 2016 Addendum.[2] Information on saccharide contents (galactose, glucose, fructose, sucrose, maltose, lactose, and trehalose) was available for 880 of 2,222 food items (39.6%) in STFCJ. Values were determined using high-performance liquid chromatography (HPLC) for 396 items (45.0%); based on information on similar foods in STFCJ for 159 items (18.1%); based on recipes for 172 items (19.5%); and based on data from food composition database in other countries (the United States,[3] the United Kingdom,[4] and Australia[5]) for 153 items (17.4%).

Total sugar

Total sugar was defined as sum of mono- and disaccharides according to the United States Department of Agriculture (USDA).[3] Thus, total sugar content was calculated as sum of the contents of glucose, fructose, galactose, sucrose, lactose, and maltose. A comprehensive total sugar database was developed according to the following seven-step method (**Figure 2** and **Table 1**). For 880 items for which information on saccharide contents was available, total sugar contents were calculated based on the values in STFCJ (Step 1). For the remaining 1,342 items without saccharide contents, a data-gathering stepwise strategy proposed by Rand et al.[6] was used, as follows:

Step 2: Assign 0 g per 100 g of food to food items with <1 g per 100 g available carbohydrates (calculated as subtracting dietary fiber content [g per 100 g of food] from carbohydrate) (n=712).

Step 3: Assign analytical values reported in the literature (n=188).

Step 4: Use values of similar food items in STFCJ (n=166).

Step 5: Estimated based on recipes or ingredients (n=135).

Step 6: Use values from food composition database in other countries (the United States[3] [n=45], the United Kingdom[4] [n=15], and Australia[5] [n=13]).

Step 7: Assign 0 g per 100 g of food (n=68).

For Step 3, a literature search was conducted based on the Pubmed, Ovid Medline, CiNii, and Ichushi-Web (a database for Japanese papers) to identify studies reporting sugar contents in Japanese foods. The search terms used were “(sugar OR sugars OR saccharide OR saccharides OR monosaccharide OR monosaccharides OR disaccharide OR disaccharides OR glucose OR fructose

OR galactose OR sucrose OR maltose OR lactose) AND food AND (Japan OR Japanese)". The only papers written in English or Japanese were considered. References from relevant articles were also manually searched. In total, 13 papers were identified: seven based on HPLC,[7–13] one based on both HPLC and gas chromatography (GC),[14] two based on GC,[15, 16] and three based on enzymatic methods.[17–19] The papers in which data were obtained using HPLC were primarily considered. Then the papers in which data were obtained using GC or enzymatic methods were considered.[20] When multiple papers reported saccharide contents of the same food item using the same analytical method, the value from the newest paper was used since sugar contents of food items might change over time due to selective breeding, progress in cultivate techniques, and reformulation of commercial products.[21, 22] Consequently, values reported in three[7, 9, 15] of 13 candidate papers were not used. For each food item, mean or median value shown in a paper was used as a representative value.

Added and free sugar

Added sugar was defined as sugars and syrups added to food during processing or preparation excluding naturally occurring sugars in foods.[23] Free sugar was defined according to the World Health Organization definition as all mono- and disaccharides added to foods and beverages by the manufacture, cook or consumer, and sugars naturally presenting in honey, syrups, fruit juices, and fruit juice concentrate.[24] Contents of added sugar in all food items were determined using a published stepwise method[25] based on contents of total sugar and saccharides, as follows (**Table 2**):

Step 1: Assign 0 g per 100 g of food to food items with 0 g total sugar (n=843).

Step 2: Assign 0 g per 100 g of food to no added sugar food groups (e.g., plain cereals [such as grains, breads, pastas, rice, and flours], plain nuts and pulses, fresh fruits and vegetables, fresh meat and seafood, egg, non-sweetened dairy products, fats and oils, 100% fruit and vegetable juices, non-sweetened coffee and tea, and non-sweetened alcoholic beverages) (n=818).

Step 3: Assign values of total sugar to 100% added sugar food groups (e.g. sugar and syrups; processed meats; confectioneries not containing fruits, dairy products, and chocolates; soft drinks except for fruit drinks; and stock powder) (n=249).

Step 4: Calculated based on standard recipes available in STFCJ for food items for whose ingredients values were all assigned in Steps 1–3 (n=62).

Step 5: Calculated based on unsweetened variety (n=15).

Step 6: Estimated from content of each saccharide (e.g., sweetened dairy products and confectioneries containing dairy products) (n=25).

Step 7: Use values from food composition database in other countries (Australia[25] [n=1] and Denmark[26] [n=2]).

Step 8: Calculated based on common recipes or ingredient lists (n=118).

Step 9: Calculated based on standard recipes available in STFCJ to food items for whose ingredients values were assigned in Step 5–8 (n=53).

Step 10: Assign a half of total sugar content (n=26).

Step 11: Assign 0 g per 100 g of food (n=10).

Free sugar contents were estimated as sum of added sugar contents and total sugar contents from fruit juices.

REFERENCES

1. Science and Technology Agency. *Standard Tables of Food Composition in Japan - 2015 - (Seventh Revised Edition)*. Tokyo, Japan: National Printing Bureau; 2015 (in Japanese).
2. Science and Technology Agency. *Standard Tables of Food Composition in Japan - 2015 - (Seventh Revised Edition) Addendum - 2016 -*. Tokyo, Japan: National Printing Bureau; 2016 (in Japanese).
3. US Department of Agriculture. *Composition of Foods Raw, Processed, Prepared USDA National Nutrient Database for Standard Reference, Release 28 (2015).* Maryland, the US: US Department of Agriculture [Internet]; 2016 [cited 2018 Jan 25]. Available from: https://www.ars.usda.gov/ARSUserFiles/80400525/Data/SR/SR28/sr28_doc.pdf.
4. Public Health England. *McCance and Widdowson's The Composition of Foods Integrated Dataset (CoFIDS).* Public Health England [Internet]; 2015 [cited 2018 Jan 25]. Available from: https://www.gov.uk/government/publications/composition-of-foods-integrated-dataset-cofid.
5. Food Standards Australia New Zealand. *NUTTAB2010 Food Composition Database.* Food Standards Australia New Zealand [Internet]; 2011 [cited 2018 Jan 25]. Available from: http://www.foodstandards.gov.au/science/monitoringnutrients/nutrientables/Pages/default.aspx.
6. Rand WM, Pennington JAT, Murphy P, Klensin JC. *Compiling Data for Food Composition Data Bases.* Tokyo, Japan: United Nations University Press; 1991.
7. Yasui T, Furukawa T, Hase S. High Performance Liquid Chromatographic Determination of Saccharides in Dairy Products. *Nippon Shokuhin Kogyo Gakkaishi.* 1980;27(7):358-362 (in Japanese).
8. Yamazawa K. The Free Sugars of Arrowheads. *Bull Tokai Women's Jr Coll.* 1982;8:21-29 (in Japanese).
9. Tsuji M, Komiyama Y. Sugar composition and water activity of confectionary in Yamanashi prefecture and the other prefecture. *Rep Inst Wine Food Technol Prefect.* 1986;(18):75-85 (in Japanese).
10. Ishiwata H, Yano S, Okuda S, Kotani T, Tsuji K. Analysis of contents of fatty acids, sterols,

saccharides and dietary fibers in confectioneries on the market. *Nippon Eiyo Shokuryo Gakkaishi.* 1988;41(5):415-422 (in Japanese).

11. Yoshikawa K, Murata Y, Murao R, Inagaki K, Terashita T, Shishiyama J. Preparation of Tables of Sugars in Food and Survey of Daily Sugar Intake. *Food Hyg Saf Sci.* 1993;34(1):55-62 (in Japanese).
12. Masuda T, Kawano A, Kitahara K, Nagashima K, Aikawa Y, Arai S. Quantitative determination of sugars and myo-inositol in citrus fruits grown in Japan using high-performance anion-exchange chromatography. *J Nutr Sci Vitaminol (Tokyo).* 2003;49(1):64-68.
13. Yu X, Xu B, Sawamura M. Determination of Sugar and Organic Acid Contents in Yuzu Juices from Different Districts of Japan by HPLC. *J Japanese Soc Hortic Sci.* 2004;73(3):293-298 (in Japanese).
14. Tanaka H, Date C, Okazaki K, *et al*. Joyo syokuhin-chu no shoto, kato, budoto, bakugato, nyuto, sorubitoru ganyuryo ni kansuru kenkyu -torui syokuhin seibun-hyo－ (The contents of sucrose, fructose, glucose, maltose, lactose and sorbitol in daily foods -sugar composition of daily foods-). *Japanese J public Heal.* 1983;30(12):607-618 (in Japanese).
15. Ishiguro K, Date Y. Sugars Content of Drinks and Ice-Cakes on Market Separative Determination of Glucose, Fructose and Sucrose by Gaschromctography. *Japanese J Nutr Diet.* 1980;38(1):71-77 (in Japamese).
16. Ishii Y. Sugar Components of Some Dry Fruits. *Nippon Eiyo Shokuryo Gakkaishi.* 1983;36(1):53-55 (in Japanese).
17. Ming Chang W, Chin Shu C. Variation of Sugars Distribution in Various Parts of Pitaya (Hylocereus undatus Britt. et Rose). *Food Preserv Sci.* 1998;24(6):17-22.
18. Yamamoto S, Taniguchi H, Sarukura N, Tsao H, Tseng A, Takeichi H. Development of a Food Composition Database of Monosaccharides and Disaccharides in Sweet Snacks and Beverages. *J Japan Diet Assoc.* 2009;52(4):314-317 (in Japanese).
19. Takeichi H, Wakikawa N, Taniguchi H, *et al.* Concentrations of Monosaccharides and Disaccharides in Commercial Sweet Snacks. *J Japan Diet Assoc.* 2010;53(1):23-26 (in Japanese).
20. Greenfield H, Southgate D. *Food Composition Data: Production, Management and Use.*

Rome, Italy: Food and Agriculture Organization of the United Nations [Internet]; 2003 [cited 2018 Jan 25]. Available from: http://www.fao.org/docrep/008/y4705e/y4705e00.htm.

21. Yasui A. Outline of Standard Tables of Food Composition in Japan - 2015 - (Seventh Revised Edition). *Japanese J Nutr Diet.* 2016;74(4):81-88 (in Japanese).

22. US Department of Agriculture. *USDA Database for the Added Sugars Content of Selected Foods, Release 1.* US Department of Agriculture [Internet]; 2016 [cited 2018 Jan 25]. Available from: https://www.ars.usda.gov/northeast-area/beltsville-md/beltsville-human-nutrition-research-center/nutrient-data-laboratory/docs/usda-database-for-the-added-sugars-content-of-selected-foods-release-1/.

23. US Department of Agriculture. *What are added sugars?* US Department of Agriculture [Internet];2016 [cited 2018 Jan 25]. Available from: https://www.choosemyplate.gov/what-are-added-sugars.

24. World Health Organization. *Guideline: Sugars Intake for Adults and Children.* Geneva, Switzerland: WHO; 2015.

25. Louie JCY, Moshtaghian H, Boylan S, *et al*. A systematic methodology to estimate added sugar content of foods. *Eur J Clin Nutr*. 2015;69(2):154-161.

26. National Food Institute Technical University of Denmark. *Frida Food Data Version 2* [Internet]; 2016 [cited 2018 Jan 25]. Available from: http://frida.fooddata.dk/?lang=en.
